# Supplementary material for: Oral Gel Formulation of Cotinus coggygria Scop. Stem Bark Extract: Development, Characterization, and Therapeutic Efficacy in a Rat Model of Aphthous Stomatitis
Source: Pharmaceutics. 2025 Oct 2;17(10):1293. doi: 10.3390/pharmaceutics17101293 (PMC12566690; doi:10.3390/pharmaceutics17101293)
Supplement: Supplementary file 1 [file pharmaceutics-17-01293-s001.zip › pharmaceutics-3870372-supplementary.pdf]

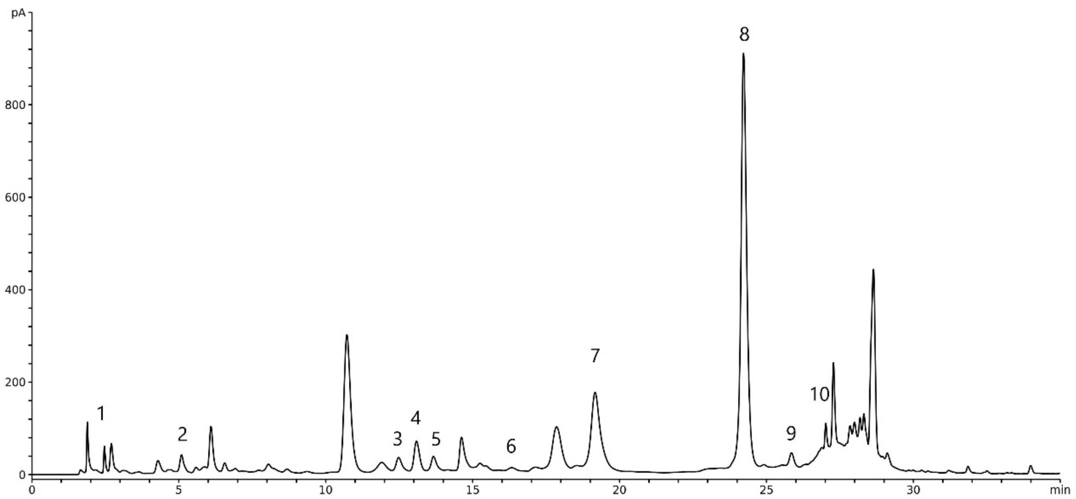

Figure S1. Chromatogram of Cotinus coggygria bark methanolic extract recorded at 260 nm. 1) gallic acid, 2) chlorogenic acid, 3) rutin, 4) hyperoside, 5) isoquercitrin, 6) quercitrine, 7) fisetin, 8) sulphuretin, 9) ellagic acid, 10) quercitin.
